# Annotations

**Published:** 1901-10-19

**Authors:** 


### ANNOTATIONS.

### Army Medical Service Reform.

Whatever may be done to render the Army Medical Department more efficient no one can doubt that the first thing is to get the men. No scheme of reform can be of the slightest practical utility so long as doctors cannot be obtained. A suggestion has again been made which we put forward some time ago, namely, that the Government should itself train its own medical officers, as has been done with great success in Germany for many years back. Speaking on Monday at the post-graduate college of the West London Hospital Sir William MacCormac said that he wished some means could be devised for removing the Army Medical School from its isolated position at Netley, and bringing its staff and its students to London. He believed that if a great military hospital and school of the most modern and complete type were established in London, and a body of teachers, similar to that of the Kaiser Wilhelm Institute in Berlin, were appointed in connection with it, a more wide-reaching influence would be exerted for the improvement and advancement of the medical department of our Army than any other single measure could accomplish. This brings us round to the very thing which we suggested. This institute in Berlin is, among other duties, charged with the training of a number of youths, selected by examination and sometimes by nomination, who are given a complete medical education on very favourable terms on the one condition that when qualified they shall enter the Army Medical Service. By this means the Government secures a constant supply of young medical men of the best possible stamp who not only form an admirable Medical Corps while they remain attached to the Army, but act afterwards as a useful medical reserve, always ready for employment in the case of national emergency. We must not quote the example of Germany, and the benefits of the Kaiser Wilhelm Institute, without recognising that the function of this institute is not merely that of a post-graduate school, but of a school for the education of medical men at Government expense for Government service.

### Small-pox and the Seasons.

The occurrence of an outbreak of small-pox at the present time of year, which is out of the common course, is a matter of some interest both in regard to immediate prospects, and to the outlook for the future. The seasonal curve of small-pox mortality is a simple one, that is to say it has one rise and one fall per annum. Taking the mean of a large number of years the maximum mortality occurs between January and May, when it rapidly falls, the minimum being reached in September. From this point it gradually rises again through October and November, and runs up quickly in December to its maximum in January and February. The fact then that the present outbreak began at the bottom of the curve, that is at the time when under normal conditions the tendency to small-pox is slight, may by some be taken to indicate that it will be easily dealt with. We do not, however, think that such a view is quite tenable in the light of past experience. When we find an infectious disease gradually dying away at a certain season, year after year, notwithstanding that during the preceding months of its maximum prevalence the dissemination of its infection must have been at the highest, it is clear that there must be at work some influence, we know not what, but something connected with the season, to account for the phenomena, and we cannot but believe that an infection accidentally imported during that period of decreasing prevalence is comparatively unlikely to extend. When, however, we find that at a given season, as also happens every year, the disease begins to spread, notwithstanding that the foci of infection must be at the minimum, we have to admit that there is some hidden influence at work to cause this result, and again in our ignorance of its exact nature we have to describe this as "seasonal"—a something which makes infection at such season of the year more potent for evil than at other times. Now, to apply all this to small-pox. The present outbreak has occurred at the very bottom of the curve when, under ordinary circumstances, small-pox is at the minimum. So far so good. Evidently the season favours our efforts to suppress it, and for the present we may succeed. But the infection which is now being spread abroad by these cases comes into operation at the very season of the year at which, as the natural history of the disease shows, a very little infection always goes a long way. Hence we must not be misled by the fact that this outbreak has so far shown no marked tendency to become epidemic. What we have to think of is what will happen in the early spring. We know that the seed has been sown, and not until harvest time arrives can we be sure that it has not taken root.

### Infectious Colds.

Many observations have been made and many experiments undertaken to satisfy the medical mind in regard to a thing that most people outside our profession have always taken for granted—namely, that "colds" are "catching." That they are catching no one now doubts. It is recognised by those who have the management of sanatoria for consumptives that one of the great disadvantages attending the visits of friends is that "colds" are introduced, much to the detriment of the patients. Of course every lady knows that "sniffly" people are as well avoided when social functions are on hand, and now a serious question is being asked—namely, Should a doctor visit his patients when suffering from "cold"? In the ordinary daily life of those who go out into the world and take their chance of all infections, who ride in trains and omnibuses and even go to church, the opportunities of infection are so many that it is probable that one more is of no moment. But to the chronic invalid shut off rigorously from external influences, the visit of a catarrhal person may undoubtedly be productive of injurious results. If ever this view should be seriously taken by the public—and they are kittle cattle to shoe, that same public, no one knows what fancy they will take up next—the matter might become a very anxious one, not only for medical men but for those Sick Insurance Societies which profess to give so much a week to medical men while incapacitated by illness. What is meant by incapacity?

